# Characterization of a microglia-specific humanized P2X7 receptor knock-out mouse line: Implications for translational psychoneuroimmunology

**DOI:** 10.1192/j.eurpsy.2022.937

**Published:** 2022-09-01

**Authors:** L. Urbina Trevino, I.-A. Von Mücke-Heim, J. Deussing

**Affiliations:** 1 Max-Planck-Institute of Psychiatry, Molecular Neurogenetics, Munich, Germany; 2 Max-Planck-Institute of Psychiatry, Translational Research In Psychiatry, Munich, Germany

**Keywords:** Genetically engineered mouse models (GEMMs), chronic stress and depression, human P2X7 receptor, translational neuropsychiatry

## Abstract

**Introduction:**

Depression is a common psychiatric disorder and chronic stress is considered its main environmental risk factor. Recently, immune processes including adenosine triphosphate mediated P2X7 receptor (P2X7R) signalling via microglia and macrophages (M/Ms) were found to play a critical role in depression genesis, by linking environmental stress to depression biology and symptoms.

**Objectives:**

To characterize the role of human P2X7R (hP2X7R) in psychosocial and immune stress conditions, both in vitro and in vivo.

**Methods:**

Several, custom designed mouse lines expressing the loxP-flanked, hP2X7R-sequence in the murine P2X7R locus were established. In addition, these mice possess a Cre-sensitive reporter and express a Cre recombinase fused to a mutant estrogen receptor ligand-binding domain in M/Ms. This enables conditional, tamoxifen-inducible hP2X7R inactivation and simultaneous tdTomato expression. First, we established primary microglia cell cultures and characterized them at baseline and following immune stimulation. Next, we performed behavioural assessment of hP2X7R^wt^ and microglia-specific hP2X7R^KO^ mice following chronic psychosocial stress. Last, we developed a novel in vivo two-photon microscopy (TPM) approach by use of frontolimbic cranial windows.

**Results:**

Primary hP2X7R^KO^ microglia displayed significantly lower IL-1β production, increased survival and decreased morphological activation upon immune stimulation. Although hP2X7R^KO^ mice showed a significant increase of locomotor activity at baseline, there was no impact on anxiety- and depressive-like phenotypes. Longitudinal in vivo TPM enabled morphometric characterization of cortical M/Ms over several weeks.

**Conclusions:**

Our results illustrate the great potential of this humanized mouse line for translational psychiatry. In the future, this system could proof useful to evaluate immunomodulatory approaches in chronic stress and depression.

**Disclosure:**

No significant relationships.

